# Expression of benzoyl-CoA metabolism genes in the lignocellulolytic host *Caldicellulosiruptor bescii*

**DOI:** 10.1186/s13568-019-0783-8

**Published:** 2019-05-04

**Authors:** Kyle Sander, Meredith Yeary, Kristina Mahan, Jason Whitham, Richard J. Giannone, Steven D. Brown, Miguel Rodriguez, David E. Graham, Bertrand Hankoua

**Affiliations:** 1 Bredesen Center for Interdisciplinary Graduate Research and Education, University of Tennessee, Knoxville, TN USA; 2 Department of Chemical and Biomolecular Engineering, University of Tennessee, Knoxville, TN USA; 3 Biosciences Division, Oak Ridge National Laboratory, Oak Ridge, TN USA; 4 Chemical Sciences Division, Oak Ridge National Laboratory, Oak Ridge, TN USA; 5 College of Agriculture, Science and Technology, Delaware State University, Dover, DE USA; 6 Present Address: Sandia National Laboratory, Livermore, CA USA; 7 Present Address: Becton Dickinson Diagnostics, Sparks Glencoe, MD USA; 8 Present Address: LanzaTech, Skokie, IL USA; 9 Present Address: Department of Bioengineering, University of California, Berkeley, Berkeley, CA USA

**Keywords:** *Caldicellulosiruptor bescii*, Aromatic, Benzoyl-CoA, Heterologous expression

## Abstract

Genes responsible for the anaerobic catabolism of benzoate in the thermophilic archaeon *Ferroglobus placidus* were expressed in the thermophilic lignocellulose-degrading bacterium *Caldicellulosiruptor bescii*, as a first step to engineering this bacterium to degrade this lignin metabolite. The benzoyl-CoA ligase gene was expressed individually, and in combination with benzoyl-CoA reductase and a putative benzoate transporter. This effort also assessed heterologous expression from a synthetically designed operon whereby each coding sequence was proceeded by a unique *C. bescii* ribosome binding site sequence. The *F. placidicus* benzoyl-CoA ligase gene was expressed in *C. bescii* to produce a full-length protein with catalytic activity. A synthetic 6-gene operon encoding three enzymes involved in benzoate degradation was also successfully expressed in *C. bescii* as determined by RNA analysis, though the protein products of only four of the genes were detected. The discord between the mRNA and protein measurements, especially considering the two genes lacking apparent protein abundance, suggests variable effectiveness of the ribosome binding site sequences utilized in this synthetic operon. The engineered strains did not degrade benzoate. Although the heterologously expressed gene encoding benzoyl-CoA ligase yielded a protein that was catalytically active in vitro, expression in *C. bescii* of six benzoate catabolism-related genes combined in a synthetic operon yielded mixed results. More effective expression and in vivo activity might be brought about by validating and using different ribosome binding sites and different promoters. Expressing additional pathway components may alleviate any pathway inhibition and enhance benzoyl-CoA reductase activity.

**Electronic supplementary material:**

The online version of this article (10.1186/s13568-019-0783-8) contains supplementary material, which is available to authorized users.

## Introduction

Consolidated bioprocessing (CBP) is projected to be an inexpensive route to renewably produce bioethanol from lignocellulosic biomass (Lynd et al. 2005, 2008). *Caldicellulosiruptor bescii* has emerged as a promising candidate microbial biocatalyst to achieve this (Chung et al. 2014), and it is actively being engineered to produce a more effective consolidated bioprocessing host organism. It is well suited for this, as it natively contains the ability to solubilize lignocellulosic biomass, using its array of secreted CAZy enzymes (Blumer-Schuette et al. 2014) and novel biomass digestion strategy, employing large, thermostable cellulolytic enzymes that contain multiple catalytic domains (Brunecky et al. 2013; Blumer-Schuette et al. 2014). *C. bescii* is an anaerobic thermophile that grows optimally at 78 °C and ferments a wide variety of carbohydrates, including those it solubilizes from lignocellulosic biomass (Hamilton-Brehm et al. 2010). While *C. bescii* can also solubilize lignin components (Kataeva et al. 2013), it cannot catabolize the resulting soluble constituents and decomposition products. The successful engineering of lignin monomer catabolism in *C. bescii* (or other such CBP biocatalysts) would substantially increase whole-biomass conversion in a CBP process and improve strain resistance to inhibitors released from biomass.

Organisms that catabolize lignin monomers and other aromatic species initially modify these molecules using specialized pathways with narrow and non-overlapping specificity. These intermediates are transformed, or ‘funneled’ to one of a few common metabolic intermediates, including benzoyl-Coenzyme A in anaerobic microorganisms (Harwood et al. 1998). These common intermediate metabolites then enter subsequent pathways where the aromatic ring portions are dearomatized and cleaved in energy-intensive reactions. Intermediates are further metabolized to acetyl-CoA in a pathway which resembles the β-oxidation pathway for fatty acids (Carmona et al. 2009). For every molecule of benzoyl-CoA processed, two molecules of acetyl-CoA are produced, which can then be utilized in other metabolic reactions. In anaerobic organisms, dearomatization and subsequent degradation reactions are carried out on aromatic molecules activated with CoA moieties through a thioester bond. This CoA-thioester reduces the resonance in aromatic moieties by drawing electrons away from otherwise stable resonance structures. This ultimately reduces the energetic demand of aromatic ring reduction (Fuchs et al. 2011).

One such organism able to catabolize aromatic molecules is the thermophilic archaeon *Ferroglobus placidus*, which grows optimally at 85 °C. It is able to grow on benzoate using Fe(III) as a terminal electron acceptor (Holmes et al. 2011; Tor and Lovley 2001). *F. placidus* synthesizes benzoyl-CoA as a metabolic intermediate when catabolizing several different aromatic chemicals (Holmes et al. 2012; Schmid et al. 2016). It utilizes an ATP-dependent benzoyl-CoA ligase (BCL) (Schmid et al. 2016) to add a CoA moiety to benzoate, and a type I benzoyl-CoA reductase (BCR) (Boll et al. 2014; Smith et al. 2015; Schmid et al. 2016) to enable the first, and most energetically demanding, reactions to reduce and break the aromaticity of benzoyl-CoA. Both enzymes are ATP dependent, coupling the hydrolysis of ATP to what are otherwise thermodynamically unfavorable reactions (Boll et al. 2014; Schmid et al. 2015).

Other organisms not able to natively catabolize aromatic species have been successfully engineered to do so. *E. coli,* heterologously expressing eight catabolic genes and one transport gene form *Pseudomonas putida,* gained the ability to grow on protocatechuate and 4-hydroxybenzoate (Clarkson et al. 2017), two common ‘funneling’ intermediates in aerobic lignin monomer catabolism. Similarly, a strain of *Azoarcus* CI B made deficient in its otherwise native ability to catabolize benzoate, was phenotypically rescued by heterologous expression of a complete benzoate degradation pathway (Zamarro et al. 2017).

Heterologous expression of genes in *C. bescii* generally comprises the expression of one or two genes simultaneously (e.g. a catabolic enzyme and a selection marker), each expressed from a separate promoter (Yang et al. 2009; Chung et al. 2014; Chung et al. 2015a, b, c, d). In addition to taking initial steps toward enabling lignin monomer and aromatic catabolism, another primary objective of this work was to assess *C. bescii* heterologous expression of multiple genes from a single transcript, employing ribosome binding sites of the host upstream of each gene to maximize protein expression and confer much more complex biological functions. Efforts herein constitute the first steps toward expression in *C. bescii* of the full aromatic catabolism pathway(s) in *F. placidus* linking aromatic catabolism to acetyl-CoA production.

## Materials and methods

### Construction of *C. bescii* heterologous expression strains

Heterologous expression strains were constructed in *C. bescii* JWCB018. *C. bescii* JWCB018 is a mutant derivative of the *C. bescii* wildtype strain *C. bescii* DSMZ 6725 that has been engineered for uracil auxotrophy and deficiency in M. CbeI restriction/methylation system (Chung et al. 2013a, b). *C. bescii* JWCB018 was a generous gift from, and is also available upon request from the laboratory of Janet Westpheling of the Department of Genetics at the University of Georgia, and the base wildtype strain *C. bescii* DSMZ 6725 is available through DSMZ. Two expression plasmids were constructed and transformed into *C. bescii* strain JWCB018 (Additional file 1: Figures S1 and S2, Tables S2 and S3). Plasmids were constructed using plasmid pJGW07 as the vector backbone (Chung et al. 2013a, b). Insert components were generated and cloned from synthesized dsDNA fragments (Integrated DNA Technologies, Inc. Coralville, IA), or amplifications products thereof. The codon-optimized gene sequences have been deposited into GenBank and can be retrieved at the following accession numbers: Ferp_1370_codon_optimized; MK294010, Ferp_1044_codon_optimized; MK294011, Ferp_1184_codon_optimized; MK294012, Ferp_1185_codon_optimized; MK294013, Ferp_1186_codon_optimized; MK294014, Ferp_1187_codon_optimized; MK294015. Plasmid pJOT1 expressed a codon-optimized, his-tagged copy of the Ferp_1044 gene, encoding for a Benzoyl-CoA Ligase (Holmes et al. 2012; Schmid et al. 2015) (Additional file 1: Figure S1), using a previously demonstrated *C. bescii* promoter natively found upstream of an S-layer protein (Athe_2303). This plasmid (Additional file 1: Table S2) was transformed into strain JWCB018 (Chung et al. 2013a, b) to generate strain JWCB018 pJOT1 (Additional file 1: Table S3). A second expression plasmid, pJOT2, contained a synthetic operon containing six genes, each preceded by a native *C. bescii* ribosome binding site (Additional file 1: Figure S2). Ribosome binding sites were designated as the 40 bp upstream of highly expressed *C. bescii* genes identified in a previous study of protein expression in the *Caldicellulosiruptor* genus (Blumer-Schuette et al. 2012). The individual gene ribosome binding site regions chosen for placement upstream of each gene are indicated in Additional file 1: Figure S2. Ferp_1184-Ferp_1187 encode for the four subunits of Benzoyl-CoA reductase, and Ferp_1370 is annotated as a benzoate transporter in the TIGRFAMs database. These genes, along with the before-mentioned benzoyl-CoA ligase, were codon optimized, arranged in a single transcriptional unit, and expressed from a single transcript also driven by the Athe_2303 promoter (Additional file 1: Figure S2, Table S2). This plasmid was transformed into strain JWCB018 to generate strain JWCB018 pJOT2 (Additional file 1: Table S3). An empty vector containing no expression insert, pJGW07, was also transformed into strain JWCB018, generating strain JWCB018 pJGW07, and subsequently used as an empty vector control in growth and activity experiments. Plasmid constructs and expression cassettes therein were validated by PCR, restriction digest, and Sanger sequencing of insert regions.

### Growth conditions and medium used

*Caldicellulosiruptor bescii* strains were grown in 50 mL LOD medium (Farkas et al. 2013) in serum bottles of total volume of 135 mL. In media containing sodium benzoate, 80 mM MOPS (3-(N-morpholino)propanesulfonic acid) buffer (part# BP308-500, Fisher Scientific, Hampton, NH) was added, and resazurin was omitted as it was found to interfere with UV detection of sodium benzoate. Fermentations were carried out at 75 °C shaking at 200 rpm. Growth was monitored by optical density at 680 nm as done previously (Farkas et al. 2013).

### HPLC determination of benzoate in medium supernatants

Benzoate present in media supernatants was determined using an Agilent 1290 Infinity HPLC and associated UV detector equipped with an Agilent Zorbax C18 Eclipse Plus reversed phase column (959757-902K, Agilent, Santa Clara, CA). The mobile phase used for elution was 79% monopotassium phosphate (pH = 2.5) and 21% acetonitrile. 20 μL of sample was injected into the column at a mobile phase flowrate of 1.5 mL/min. The column was maintained at 30 °C. Dilutions of sodium benzoate in LOD medium were used as standards to monitor retention time of sodium benzoate, and to generate a standard curve for quantitation. Integrated peak areas of absorbance at 230 nm was used to quantify benzoate, using the absorbance value at 360 nm as the reference.

### Western blot and benzoyl-CoA activity assay

50 mL batch cultures of JWCB018 pJOT1 and JWCB018 pJGW07 were grown to mid-log cell density in medium containing sodium benzoate. Mid-log cells were centrifuged at 20,000×g at 4 °C for 4 min in a Piramoon fixed-angle FiberLite rotor (ThermoScientific, Waltham, MA), snap frozen in liquid nitrogen, and stored at − 80 °C. Cell pellets were resuspended in 1 mL of CelLytic B (part # B7435, Sigma Aldrich, St. Louis, MO) and sonicated for 6 cycles at 12 W for 15 s, with 1-min rest between cycles in a Misonix 3000 Sonicator (Misonix Incorporated, Farmingdale, NY). The sonicated mixture was centrifuged for 30 min at 4 °C at maximum speed in a microcentrifuge and the supernatant was retained as clarified cell extract. This extract was used in his-antibody immunodetection (western blot) of Ferp_1044 encoded protein as well as Benzoyl-CoA activity assays.

Toward immunodetection of his-tagged protein, 5 uL of cell lysate was electrophoresed on a Novex 4–20% Tris Glycine SDS Polyacrylamide gel (part # XP04202BOX, ThermoScientific, Waltham, MA) in an XCell SureLock Mini-Cell Electrophoresis Chamber (ThermoScientific, Waltham, MA) according to manufacturer’s instructions. The gel was subsequently placed in contact with a nitrocellulose membrane and protein was transferred to the membrane according to manufacturer’s protocols at 25 V for 90 min in a transfer buffer containing 1X Novex Tris–Glycine Transfer Buffer (Part # LC3675, ThermoScientific, Waltham, MA) and 10% methanol (v/v). Immunodetection was performed on the membrane the WesternBreeze Chromogenic Kit (anti-mouse reagents, ThermoScientific, Waltham, MA) according to manufacturer’s instructions. Monoclonal Anti-Polyhistidine Antibody (mouse IgG) was used as the primary antibody (R&D Systems, Minneapolis, MN).

Benzoyl-CoA Ligase activity was assayed in clarified cell lysates at 65 °C, 70 °C, and 75 °C using a previously described assay (Kawaguchi et al. 2006), though 25 uL of the aforementioned prepared crude cell lysate was used in place of purified protein. The assay was carried out in a Beckman Coulter DU 800 UV spectrophotometer (Beckman Coulter, Brea, CA) using UV transparent cuvettes (Plastibrand, Fisher Scientific, Hampton, NH).

### RNA extraction, cDNA synthesis, and qPCR

RNA was extracted from 50 mL of mid-log grown batch culture replicates harvested and centrifuged at 20,000×*g* at 4 °C for four minutes, snap frozen in liquid nitrogen, and stored at − 80 °C. Total RNA was extracted by first incubating cell pellets in 250 µL of 20 mg/mL Lysozyme (Sigma Aldrich part number L-7651, St. Louis, MO) resuspended in SET buffer (50 mM Tris–HCl pH 8.0 50 mM EDTA, 20% w/v Sucrose) and incubated in a dry stationary bath at 37 °C for 8 min, vortexing briefly every 2 min. RNA was purified with a Qiagen RNEasy Kit according to manufacturer’s protocol (Qiagen, Hilden, Germany). RNA concentration was quantified with a Nanodrop 1000 instrument (ThermoScientific, Waltham, MA) and RNA quality was assessed via RNA Integrity Numbers (RIN) obtained with an Agilent 2100 Bioanalyzer and corresponding RNAchip (Agilent Technologies, Santa Clara, CA). cDNA was generated using a ScriptSeq II Kit (Illumina, San Diego, CA) according to manufacturer’s instructions. cDNA was diluted and used as template in qPCR reactions using Roche FastStart SYBR Green Master Mix (ThermoScientific, Waltham, MA) according to manufacturer’s instructions. Primers were designed to amplify a unique ~ 100 bp region within the CDS of each gene of interest. Primers used to quantify expression of each gene (relative to that of Athe_0001) are listed in Additional file 1: Table S1.

### Quantitative proteomic determination of unique peptides derived from benzoate catabolism enzymes

Cell biomass was collected from 50 mL of mid-log grown batch culture replicates grown in medium containing sodium benzoate (see culture conditions methods). Strains assessed were JWCB018 pJOT2 and JWCB018 pJGW07. Mid-log phase culture replicates were centrifuged at 20,000×*g* at 4 °C for 4 min and snap frozen in liquid nitrogen. Protein was isolated from cell biomass and proteomic determination was carried out as described previously (Eminoğlu et al. 2017).

## Results

### Benzoate inhibits growth of *C. bescii*

**Fig. 1:**
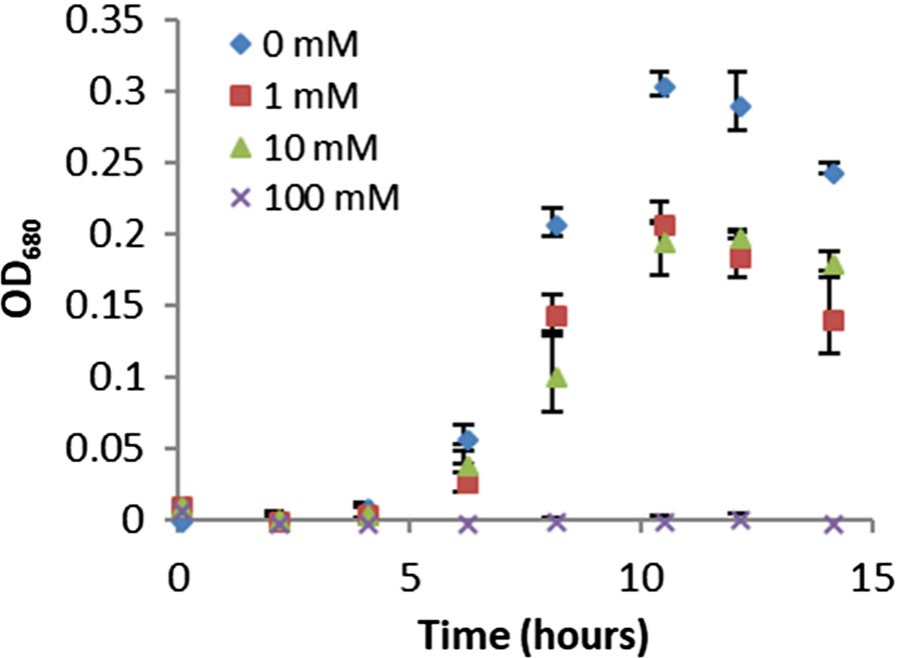
*Caldicellulosiruptor bescii* strain JWCB018 grown in increasing concentrations of sodium benzoate. Growth inhibition is observed at concentrations of 1 mM sodium benzoate and above. Error bars represent the standard deviation from analysis of 3 replicate cultures

The addition of sodium benzoate to the culture medium significantly inhibited the growth of *C. bescii* at concentrations as low as 1 mM (Fig. 1). Cultures containing benzoate yielded ~ 50% lower maximum cell density than unamended cultures. Cultures containing 10 mM sodium benzoate exhibited an average maximum growth rate which is 27% lower than unamended cultures (0.48 h^−1^ relative to 0.65 h^−1^ of unamended cultures). Benzoate was the most abundant monoaromatic compound identified in a previous anaerobic enrichment culture grown on radiolabeled lignin (Colberg and Young 1985), and it is a common food preservative with known antimicrobial activity. Therefore, benzoate accumulation during lignocellulosic decomposition could inhibit growth of this biocatalyst.

### Benzoyl-CoA ligase heterologous expression and activity

**Fig. 2:**
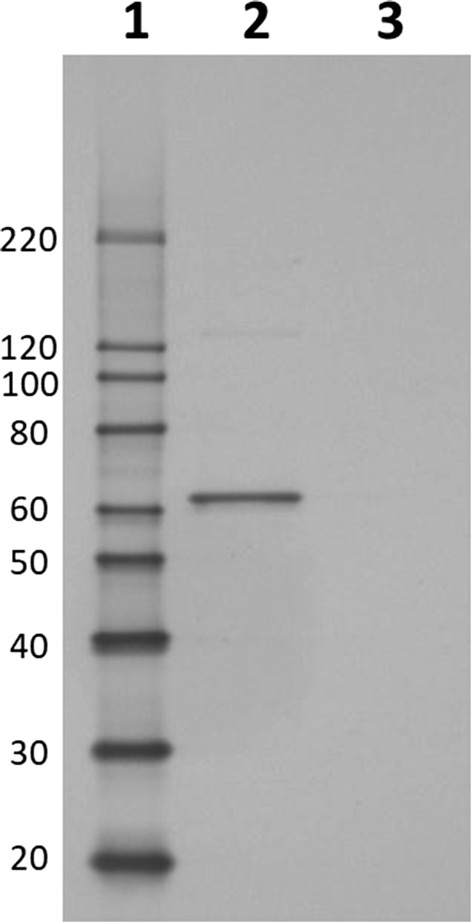
Western blot image of whole-cell lysate from *C. bescii* expression strain JWCB018 pJOT1 showing a protein product of the size expected for heterologously expressed *F. placidus* benzoyl-CoA ligase protein as the major detected product. Whole-cell lysate was prepared, electrophoresed, and probed using anti-6× histidine antibody (see methods). Lane 1: MagicMark XP Western Protein Standard (ThermoFischer Scientific, Waltham, MA) protein masses (in kDa) indicated adjacent to each band, Lane 2: Lysate from strain JWCB018 pJOT1, Lane 3: Lysate from strain JWCB018 pJGW07 (hosting an empty expression vector)

*C. bescii* strain JWCB018 pJOT1 was constructed to heterologously express a codon-optimized *Ferroglobus placidus* benzoyl-CoA ligase gene. This strain produced full length benzoyl-CoA ligase with a short affinity tag that enabled identification by Western blot analysis (Fig. 2). Furthermore, we observed benzoyl-CoA ligase activity in clarified cell extracts of this strain that was not present in a control strain that lacked the *F. placidus* benzoyl-CoA gene. Benzoyl-CoA ligase activity was detected at 65 °C (Table 1), but not when the assay was carried out at 70 °C or 75 °C (data not shown). The *C. bescii* expression platform used in this study has enabled *C. bescii* to produce ethanol (Chung et al. 2014; Chung et al. 2015a, b, c, d), detoxify furan aldehydes (Chung et al. 2015a, b, c, d), and increased cellulolytic capability (Chung et al. 2015a, b, c, d). Here we further expanded *C. bescii’s* repertoire of capabilities to activate a lignin degradation product.

**Table 1:** Benzoyl-CoA ligase activity in clarified cell extract of a *C. bescii* strain heterologously expressing a benzoyl-CoA ligase from *Ferroglobus placidus* grown at 65 °C

Strain	Expressed *F. placidus* protein	Benzoyl-CoA ligase activity (pmol × mg protein^−1^ × min^−1^)
JWCB018 pJOT1	Benzoyl-CoA ligase	1.35 ± 0.84
JWCB018 pJGW07	None (empty vector)	0.01 ± 0.01

### Expression of the benzoyl-CoA reductase and putative benzoate transport genes

**Fig. 3:**
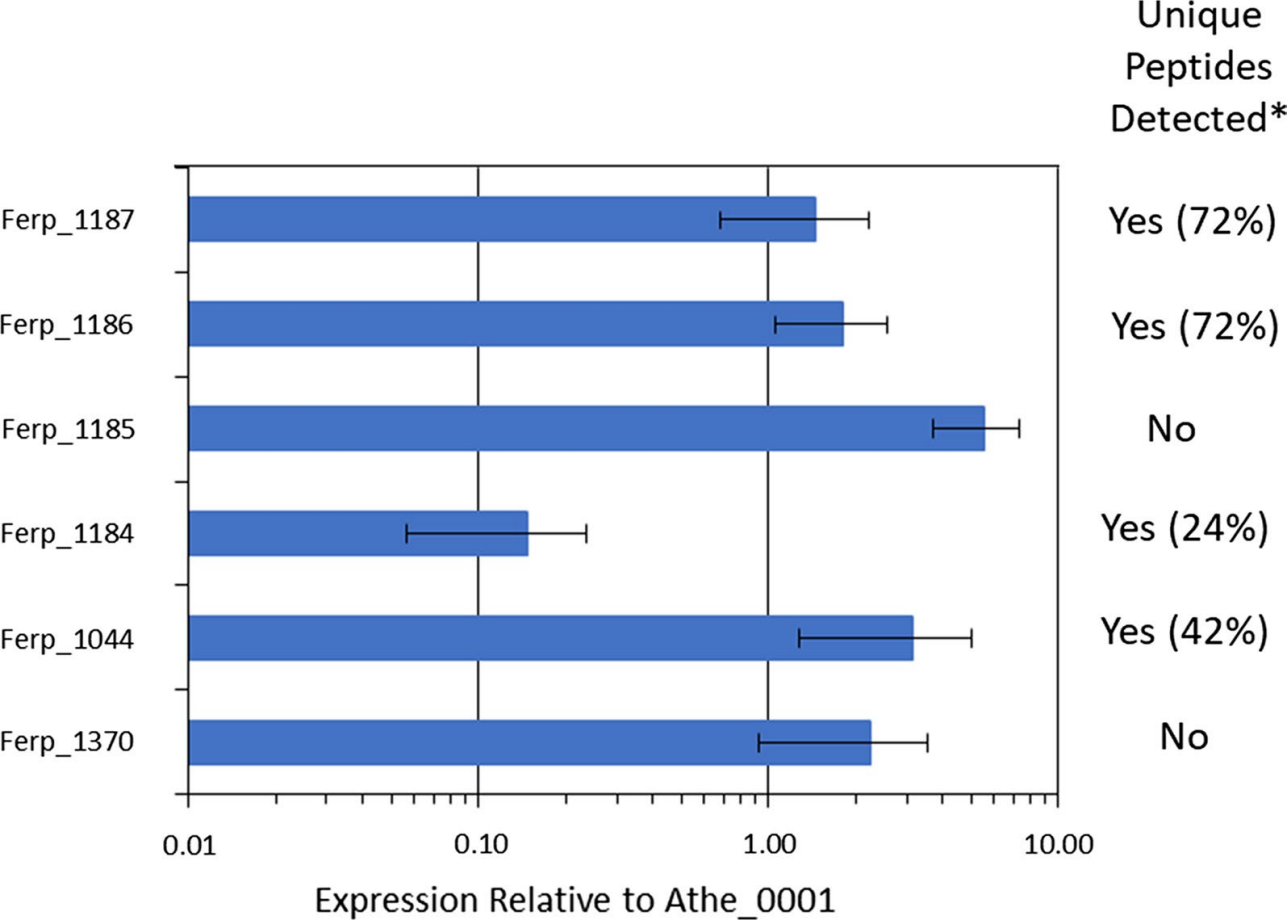
Relative expression of heterologously expressed benzoate catabolism genes in strain JWCB018 pJOT2 measured by RT-qPCR. Relative expression was calculated with respect to *dnaA*, a constitutively expressed chromosomal replication initiation protein (Athe_0001). Though all genes appeared to be transcribed, unique peptides were not identified for two proteins: Ferp_1370 (a putative benzoate transporter) and Ferp_1185 (one of four subunits of benzoyl-CoA reductase). *Heterologously expressed *Ferroglobus placidus* proteins were deemed present if normalized protein abundance was significantly (*p* value < 0.02, n = 3, log2 fold change > 1) greater in strain JWCB018 pJOT2 than in strain JWCB018 pJGW07. Numbers in parenthesis indicate percentages of each gene’s coding sequence represented by unique peptides identified through intracellular proteomics

**Fig. 4:**
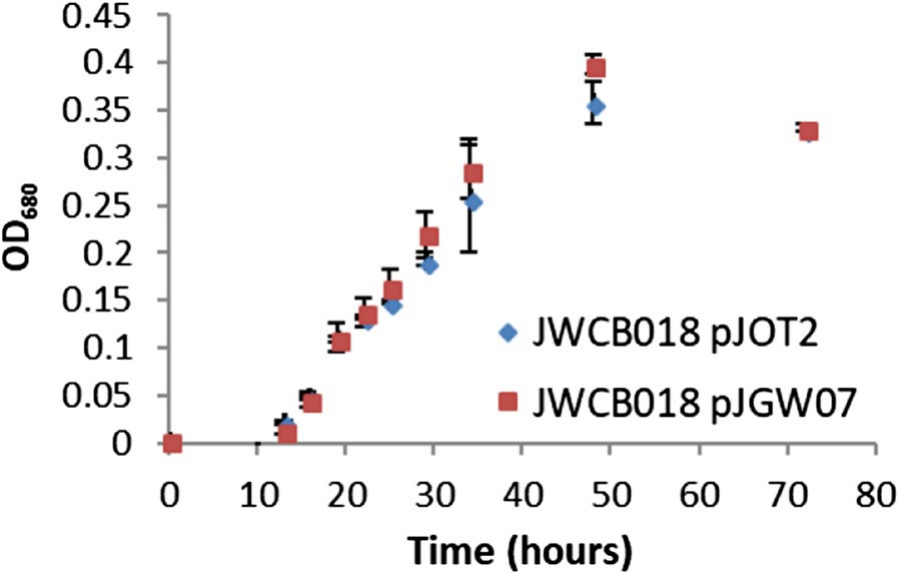
Growth of *C. bescii* strains JWCB018 pJOT2 and JWCB018 pJGW07 in defined medium (containing 5 g/L maltose) supplemented with 10 mM sodium benzoate and 80 mM MOPS buffer (initial pH = 6.8). Error bars represent the standard deviation from analysis of 3 replicate cultures

**Fig. 5:**
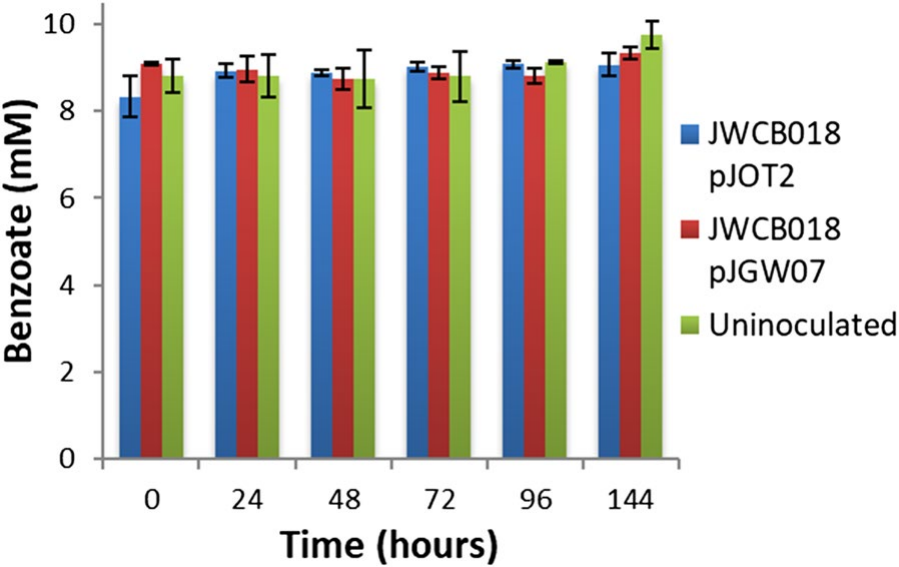
Time course of benzoate concentration in cultures of *C. bescii* grown in defined medium containing 10 mM added sodium benzoate and 80 mM MOPS buffer. Benzoate concentrations do not decrease through 144 h of fermentation in the presence of *C. bescii* strain JWCB018 pJOT2. Error bars represent the standard deviation from analysis of 3 replicate cultures

*Caldicellulosiruptor bescii* strain JWCB018 pJOT2 was constructed to express six *F. placidus* genes from a single synthetic operon whose expression was driven by the promoter of a native *C. bescii* gene encoding a highly abundant protein, the S-layer protein (Athe_2303), of *C. bescii*. These genes encoded a putative benzoate transporter, benzoyl-CoA ligase, and four subunits of benzoyl-CoA (Holmes et al. 2011, Holmes et al. 2012; Smith et al. 2015). RNAs transcribed from each gene were detected in varying abundance for all six genes by RT-qPCR (Fig. 3). However, unique peptides could only be identified from four of six genes using proteomic analysis (Fig. 3). Peptide evidence was not detected for one of the four subunits of benzoyl-CoA reductase (Ferp_1185), or for the putative benzoate transporter (Ferp_1370) encoded on the heterologous expression vector pJOT2 (Additional file 1: Figure S2). Furthermore, no in vivo catabolism of 10 mM sodium benzoate was observed during fermentation by *C. bescii* strain JWCB018 pJOT2 (Figs. 4 and 5) after 144 h of fermentation.

Variation in protein production from heterologous gene expression may have been a result of transcript instability or strengths of the different native *C. bescii* ribosome binding sites used in this heterologous expression design. We did not see a trend of decreasing mRNA abundance along the length of the transcript, suggesting incomplete transcription and/or mRNA production was not the primary cause of variable expression among the genes. There is also no noticeable correlation between protein abundance and the relative amount of mRNA expression from any of the genes, suggesting either differing strengths of ribosome binding sites employed, varying rates of protein accumulation and turnover, or mRNA degradation may be reasons for the observed variation in protein production.

## Discussion

### Relatively low activity of heterologous benzoyl-CoA ligase

Benzoyl-CoA ligase activity from this strain was found to be four orders of magnitude smaller than comparable activity measurements in other organisms which natively host and express Benzoyl-CoA ligase (Schühle et al. 2003; Kawaguchi et al. 2006), suggesting expression and/or assay conditions are far from optimal. One reason for this observed discrepancy might be that the in vitro assay conditions do not represent conditions for optimal activity. Native intracellular levels of ATP are relatively low in *C. bescii* (Bielen et al. 2010), suggesting the primary source of phosphate cleavage derived energy is pyrophosphate and not ATP. Pyrophosphate is a product in the biochemical reaction carried out by benzoyl-CoA ligase, and carryover of this chemical in lysates used in in vitro assays may be inhibiting the activity of the heterologous benzoyl-CoA ligase. Expression of benzoyl-CoA ligase mRNA in strain JWCB018 pJOT2 was found to be 2–3 fold higher than the housekeeping gene *dnaA* (Fig. 3). Other assessments of benzoyl-CoA ligase activity have been done using lysates that were prepared from cells natively hosting this enzyme (Geissler et al. 1988; Kawaguchi et al. 2006), where elevated concentrations of added sodium benzoate are known to strongly induce expression of benzoyl-CoA ligase (Holmes et al. 2011). In this study, the *bcl* gene was expressed from a constitutive promoter (Additional file 1: Figure S1), and the sodium benzoate contained in medium used in this study noticeably inhibited *C. bescii* growth (Fig. 1). Increasing heterologous benzoyl-CoA ligase activity (and possibly that of other heterologous aromatic catabolism enzymes) in *C. bescii* may be realized by increasing production of the enzyme, increasing intracellular concentrations of ATP, and/or decreasing the intracellular pyrophosphate concentration.

### Redox considerations of benzoyl-CoA reduction

Additional genes that may be necessary for benzoate catabolism in *F. placidus* may be needed to effect benzoate catabolism in *C. bescii*. Ferp_1180 encodes a ferredoxin that is co-expressed and displays the same benzoate-driven expression patterns as other aromatic catabolism genes in *F. placidus* (Holmes et al. 2012). The benzoyl-CoA reductase is annotated as being ferredoxin dependent (Holmes et al. 2012), although reduction via this enzyme has been demonstrated using a number of different electron donors (Schmid et al. 2015). This particular ferredoxin, as well as other genes found to be co-regulated in response to benzoate (Holmes et al. 2012), may be necessary for proper functioning of this enzyme.

In other species, oxidoreductase genes are needed to re-reduce the low potential ferredoxin needed for benzoyl-CoA reductase mediated reduction (Carmona et al. 2009). Homologs of the Ferp_1033–Ferp_1034 genes, encoding a 2-oxoglutarate ferredoxin oxidoreductase (Holmes et al. 2012; Boll et al. 2014), were shown to be important to this recycle, and supplying reduced ferredoxin, in *Thauera aromatica* (Dörner and Boll, 2002). A gene encoding one subunit of 2-oxoglutarate ferredoxin oxidoreductase in *F. placidus* was found to have increased expression when grown on aromatic substrates (Holmes et al. 2012). The expression of 2-oxoglutarate ferredoxin oxidoreductase from *F. placidus* (Ferp_1033–Ferp_1034) may also serve to increase benzoyl-CoA reductase activity in vivo in *C. bescii*.

It was found that the heterologous putative benzoate transporter was unable to be expressed from the synthetic operon construct in strain JWCB018 pJOT2. As such, another reason no benzoate degradation was observed in this strain (Fig. 5) was that this strain was unable to import benzoate. The effectiveness of the protein product from the gene Ferp_1370 has not been tested for its ability to facilitate benzoate import into a bacterium.

## Additional file

**Additional file 1: Figure S1.** Diagram of plasmid pJOT1 used to heterologously express a codon-optimized Ferp_1044 gene from *Ferroglobus placidus*, encoding a benzoyl-CoA ligase, in *C. bescii*. **Figure S2.** Diagram of plasmid pJOT2 used to heterologously express codon-optimized genes from *Ferroglobus placidus* encoding a benzoyl-CoA ligase (Ferp_1044), a benzoyl-CoA reductase (Ferp_1184–Ferp_1187), and a putative benzoate transporter (Ferp_1370). Unique ribosome binding site regions (~ 40 upstream basepairs) were identified from highly transcribed *C. bescii* genes (Blumer-Schuette et al. 2012) and placed upstream of each gene. **Table S1.** Primers used in this study. **Table S2.** Plasmids used in this study.

## Abbreviations

bp: basepairs (of DNA)
qPCR: quantitative polymerase chain reaction
PCR: polymerase chain reaction
CoA: coenzyme A
EDTA: ethylenediaminetetraacetic acid
CBP: consolidated bioprocessing
BCL: benzoyl-coenzyme A ligase
DOE: U.S. Department of Energy
