# Supplementary material for: Expression of benzoyl-CoA metabolism genes in the lignocellulolytic host Caldicellulosiruptor bescii
Source: AMB Express. 2019 May 4;9:59. doi: 10.1186/s13568-019-0783-8 (PMC6500515; doi:10.1186/s13568-019-0783-8)
Supplement: Supplementary file 1 — Additional file 1: Figure S1. Diagram of plasmid pJOT1 used to heterologously express a codon-optimized Ferp_1044 gene from Ferroglobus placidus, encoding a benzoyl-CoA ligase, in C. bescii. Figure S2. Diagram of plasmid pJOT2 used to heterologously express codon-optimized genes from Ferroglobus placidus encoding a benzoyl-CoA ligase (Ferp_1044), a benzoyl-CoA reductase (Ferp_1184–Ferp_1187), and a putative benzoate transporter (Ferp_1370). Unique ribosome binding site regions (~ 40 upstream basepairs) were identified from highly transcribed C. bescii genes (Blumer-Schuette et al. 2012) and placed upstream of each gene. Table S1. Primers used in this study. Table S2. Plasmids used in this study. [file 13568_2019_783_MOESM1_ESM.docx]

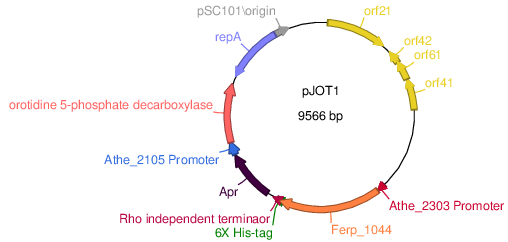


Figure S1. Diagram of plasmid pJOT1 used to heterologously express a codon-optimized Ferp_1044 gene from *Ferroglobus placidus*, encoding a benzoyl-CoA ligase, in *C. bescii*.


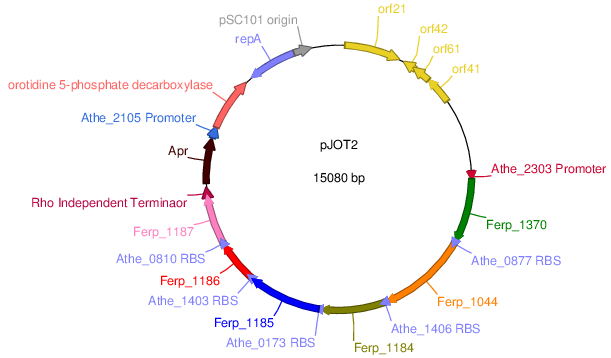


Figure S2. Diagram of plasmid pJOT2 used to heterologously express codon-optimized genes from *Ferroglobus placidus* encoding a benzoyl-CoA ligase (Ferp_1044), a benzoyl-CoA reductase (Ferp_1184 – Ferp_1187), and a putative benzoate transporter (Ferp_1370). Unique ribosome binding site regions (~40 upstream basepairs) were identified from highly transcribed *C. bescii* genes (Blumer-Schuette, Sara et al., 2012) and placed upstream of each gene.

| **Primer Name** | **Sequence** | **Purpose** |
| --- | --- | --- |
| pJGW07bb_overlap-fwd | ccctctctgacgctcagtggaacgaaaactcacgttaagggattttggtcatgag | Amplificaition of backbone of plasmid pJGW07 to clone plasmids pJOT1 and pJOT2 |
| pJGW07bb_overlap-rev | aactgtagactcgagattcccatgagcccacgaacagtaagcgcaccgttttttg | Amplificaition of backbone of plasmid pJGW07 to clone plasmids pJOT1 and pJOT2 |
| 1044f | TGTGAGAGAATAAGGAGGTGCAAATTAAAGATGATTTGGAAGAAGTGGTAC | amplification of codon optimized Ferp_1044 gene for cloning of pJOT2 |
| 1044r | CTCTACCTACCATCCCTTCGTTATTTTTTTTTAACCTCCCCTCTTCTTTC | amplification of codon optimized Ferp_1044 gene for cloning of pJOT2 |
| 1184f | AAAAAAATAACGAAGGGATGGTAGGTAGAGATGGGCGAAAACGTG | amplification of codon optimized Ferp_1184 gene for cloning of pJOT2 |
| 1184r | TTCAAATCTTCCTCCTTTCAACAAATTTCGTCATATGTAAAGATCTTCCTCACC | amplification of codon optimized Ferp_1184 gene for cloning of pJOT2 |
| 1185f | CGAAATTTGTTGAAAGGAGGAAGATTTGAAGTGACAGAAGTTAAGGTTAGACC | amplification of codon optimized Ferp_1185 gene for cloning of pJOT2 |
| 1185r | TCGTTTATTCCTCCTTCTGAATATTTTTTGTCAAGCAAGCTTTTCAAC | amplification of codon optimized Ferp_1185 gene for cloning of pJOT2 |
| 1186f | CAAAAAATATTCAGAAGGAGGAATAAACGAATGTTTGTTGGAGTAGATGTTG | amplification of codon optimized Ferp_1186 gene for cloning of pJOT2 |
| 1186r | CCTGATTTTATCCTCCTTCACAATAATATTCTAACCTTTGTAAGTTTCTAAAGCA | amplification of codon optimized Ferp_1186 gene for cloning of pJOT2 |
| 1187-bbf | AATATTATTGTGAAGGAGGATAAAATCAGGATGGTTTCAGTACAGGAGTCTG | amplification of codon optimized Ferp_1187 gene for cloning of pJOT2 |
| 1187-bbr | ggatccctcaccaaacc | amplification of codon optimized Ferp_1187 gene for cloning of pJOT2 |
| Ferp_1370f | aatcatacaaggaggtttggtgagggatccATGAGCTTGATATTCATACTGAAG | amplification of codon optimized Ferp_1370 gene for cloning of pJOT2 |
| Ferp_1370r | CTTTAATTTGCACCTCCTTATTCTCTCACATTAATGAGCTCTAACCTCTTTCC | amplification of codon optimized Ferp_1370 gene for cloning of pJOT2 |
| Ferp_1044 F | GTAGCAAACGCTTTCTCAGACTATC | qPCR verification of Ferp_1044 gene expression |
| Ferp_1044 R | ACAGCAATCATTCCGTTACTAAGAG | qPCR verification of Ferp_1044 gene expression |
| Ferp_1184 F | AAGTTAATGGCTTTAGCAAAGAGGT | qPCR verification of Ferp_1184 gene expression |
| Ferp_1184 R | TTCAACGTTCCACTCTCTTATAACC | qPCR verification of Ferp_1184 gene expression |
| Ferp_1185 F | ATTTATGCGGATACATGAGGAACTA | qPCR verification of Ferp_1185 gene expression |
| Ferp_1185 R | GCAGCAAATGTGTATAGTGAAGTTG | qPCR verification of Ferp_1185 gene expression |
| Ferp_1186 F | CGTTATATTCGCTGAATCTGAAGTT | qPCR verification of Ferp_1186 gene expression |
| Ferp_1186 R | CGTCTCTTATCTCAAGTCTTCTTGC | qPCR verification of Ferp_1186 gene expression |
| Ferp_1187 F | ACATAGTTGCTACAGGTTACGGAAG | qPCR verification of Ferp_1187 gene expression |
| Ferp_1187 R | CTCCGTATATGTAAACTGCACCTCT | qPCR verification of Ferp_1187 gene expression |
| Athe_0001 F | TTATCCTGGCACAAAGGTTATGTAT | qPCR quantification of Athe_0001 gene (relative comparator) |
| Athe_0001 R | TACTTGAGCCTAAACTCGTCTGTCT | qPCR quantification of Athe_0001 gene (relative comparator) |
| Ferp_1370 F | GGTCTTGGTGGTGGATTAACA | qPCR verification of Ferp_1370 gene expression |
| Ferp_1370 R | CAATGCTCCTATTCCCCAGA | qPCR verification of Ferp_1370 gene expression |

Table S1. Primers used in this study

| **Plasmid** | **Purpose** | **Reference** |
| --- | --- | --- |
| pJGW07 | Empty replicating expression vector used to generate empty-vector control strain JWCB018 pJGW07 | (Chung, Daehwan, Cha, Farkas, & Westpheling, 2013) |
| pJOT1 | Heterologous expression of codon-optimized Ferp_1044 gene (benzoyl-CoA ligase) in *C. bescii* | This Study |
| pJOT2 | Heterologous expression of a total of six codon-optimized genes comprising a benzoyl-CoA ligase (Ferp_1044), a benzoyl-CoA reductase (Ferp_1184 – Ferp_1187), and a putative benzoate transporter (Ferp_1370) in *C. bescii* | This Study |

Table S2. Plasmids used in this study

| **Strain Name** | **Genotype** | **Reference** |
| --- | --- | --- |
| JWCB018 | Δ*pyrF* Δ*cbeI* | (Chung, Daehwan et al., 2013) |
| JWCB018 pJGW07 | Δ*pyrF* Δ*cbeI* pJGW07 | This Study |
| JWCB018 pJOT1 | Δ*pyrF* Δ*cbeI* pJOT1 | This Study |
| JWCB018 pJOW2 | Δ*pyrF* Δ*cbeI* pJOT2 | This Study |

Table S3. Strains used in this study
